# The Role of Personality and Social Support in Health-Related Quality of Life in Chronic Kidney Disease Patients

**DOI:** 10.1371/journal.pone.0129015

**Published:** 2015-07-01

**Authors:** Norhayati Ibrahim, Sharlene S. L. Teo, Normah Che Din, Abdul Halim Abdul Gafor, Rozmi Ismail

**Affiliations:** 1 Health Psychology Programme, Faculty of Health Sciences, The National University of Malaysia (UKM), Kuala Lumpur, Malaysia; 2 Nephrology and SLE Unit, Faculty of Medicine, The National University of Malaysia (UKM), Kuala Lumpur, Malaysia; 3 School of Psychology and Human Development, Faculty of Social Sciences and Humanities, The National University of Malaysia (UKM), Bangi, Selangor, Malaysia; Mario Negri Institute for Pharmacological Research and Azienda Ospedaliera Ospedali Riuniti di Bergamo, ITALY

## Abstract

**Background:**

Chronic kidney disease (CKD) is commonly associated with various negative health outcomes. The aim of this study was to examine the influence of personality and social support on health-related quality of life in patients with chronic kidney disease. Health-related quality of life (HRQoL) is the quality of life studied in relation to health, and it provides important information of patients’ coping with their health issues.

**Method:**

Participants comprised of 200 patients experiencing various stages of chronic kidney disease. All participants completed the Short-Form 36 (SF-36), Big Five Inventory (BFI) and the Medical Outcomes Study (MOS) Social Support questionnaires.

**Results:**

Participants consisted of 108 males (54.0%) and 92 females (46.0%) with the mean age of 59.3 years (SD 14.5). Results showed that higher levels of extraversion and lower perceived affectionate social support were associated with higher physical HRQoL, whereas higher levels of neuroticism were associated with poorer mental HRQoL.

**Conclusion:**

The current study found that certain personality traits, namely extraversion and neuroticism, were found to be associated with HRQoL. In addition, affectionate social support was also associated with higher HRQoL. Therefore, special attention should be paid to the personality of CKD patients, as well as the type of social support that they have, in planning interventions to improve their health outcomes.

## Introduction

Chronic kidney disease (CKD) is an overall term that describes a wide range of disorders that affects the structure and function of the kidney [1], which is present for more than three months [2], [3]. At its worst phase, CKD can progress into end-stage renal disease. It is a major growing health issue in developed and developing countries worldwide [4], including Malaysia [3]. The prevalence in West Malaysia itself was found to be 9%, which is similar to what was reported in other Asian countries [5]. Therefore, it is crucial to study the effects of CKD as it is associated with increased hospitalisation, cardiovascular disease and mortality [6], [7], [8], [9], [10] that would significantly cause human, economic and social burdens on the nation’s health care system [3], [11]. Consequently, much research has been done in an attempt to understand the factors that influence the condition and its progression in CKD patients.

CKD not only compromises the physical health of patients but it also affects their psychological health, daily functioning, general wellbeing and social functioning, which are determinants of the patient’s quality of life [12], [13]. The World Health Organization (WHO) defined quality of life as how one perceived their position in life in taking into consideration their culture and value systems as well as its relation to their goals, expectations, standards and concerns [14]. Health-related quality of life (HRQoL) is the kind of quality of life that is impacted by health-related issues, and it can provide crucial information concerning how the patient is coping with their CKD condition [15]. Generally, CKD has a negative effect on sufferers’ HRQoL [16]. HRQoL was compromised even in the early stages of CKD [15], [17], [18]. Poorer HRQoL is also associated with higher risk of developing end-stage kidney disease, which then predicts mortality and hospitalization [8], [9], [19], [20].

Research has found some links between personality traits and certain health related outcomes [6], [21], [22], [23]. The most common personality model explored has been the Big Five personality traits, which encompassed openness, conscientiousness, extraversion, agreeableness, and neuroticism. In general, openness, extraversion and conscientiousness traits have been associated with perceived better health [21]. Specifically, it has been found that neuroticism is associated with increased risk for physical disorders including kidney disease [6], [22]. Neuroticism was also related to perceived poorer health [6], [21]. On the other hand, conscientiousness was related to better adherence to prescribed medication [6], [23]. Therefore, certain personality traits have been associated with behaviours that will lead to better HRQoL.

Social support has been found to alleviate the negative impacts of CKD on patients’ HRQoL. Social support is the complex network of how a person gets and give information and aid, as well as how they meet their emotional needs [24]. This can be acquired from family, friends, and other social networks available to the individual [21], [22]. Social support has been found to be associated with better survival, lower depression and higher compliance to medication [24], [25]. In addition, it has also been related to better immune function as well [24]. Poorer social support has been found with increased mortality [26], decreased HRQoL [26] and increased hospitalization [27].

HRQoL is significantly compromised with the presence of CKD, and it has been found that personality and social support can influence HRQoL outcomes in patients suffering from CKD. These psychosocial factors are important, as it could provide crucial information on prolonging CKD patients’ survival, as well as maintaining their quality of life. As such, this study aims to explore the relative influence of both personality traits and social support on HRQoL in patients suffering from CKD, specifically within the Malaysian context.

## Methods

### Ethical Approval

The current study was approved by the National University of Malaysia’s (UKM) Research Ethics Committee, [Ethics Approval no: NN-089-2013].

### Procedure

Upon ethics approval, participants were recruited from the Nephrology Clinic at the National University of Malaysia Medical Centre (HUKM). This cross-sectional study was conducted between October and December 2013 using purposive sampling method. All participation in this study was voluntary and participants were able to withdraw from the study at any time. Participants were given questionnaires that included the Short Form-36 (SF-36), Big Five Inventory (BFI) to assess their personality traits and the Medical Outcome Study (MOS) Social Support Survey to assess the level of their perceived social support that they are receiving. All patients who were able to understand Malay or English language were approached at the clinic while they were waiting to for their turn for treatment or to see the doctor for follow-up treatment.

Socio-demographic data and causes and stages of CKD were also recorded. These medical data were ascertained from history and clinical findings. Diabetes mellitus was diagnosed according to the 2006 WHO criteria [28]. Hypertension is defined as >140/90mmHg based on the Joint National Committee (JNC) 7 criteria [29]. Kidney stones were based on symptoms and imaging, and lupus nephritis was made by renal biopsy results and following the International Society of Nephrology/Real Pathology Society (ISN/RPS) 2003 classification [30]. All other causes (including glomerulonephritis) were classified under others, and unknown causes were also recorded. CKD stages were defined as estimated glomerular filtration rate (eGFR) of less than 90 mL/min per 1.73 m2 for at least three months using the CKD-EPI formula [2]. Albuminuria was not included in the analysis as it would have been indiscrimatory as all participants had albuminuria >300mg/24hours and were in category A3 based on the categories set by Kidney Disease: Improving Global Outcomes (KDIGO) 2012 clinical practice guidelines [2].

### Participants

A total of 230 CKD patients between the ages 18 and 80 years (mean age = 59.32; SD = 14.45) from the Nephrology Clinic at the National University of Malaysia Medical Centre (HUKM), who were experiencing stages two to five, were approached and given the questionnaires. However, only 200 of those questionnaires were completed. As such, the 30 incomplete questionnaires were excluded from the data analysis.

### Instruments

A structured questionnaire was used to obtain response from the participants. The demographic information on each participant was collected by the same questionnaire that included age, gender, level of education, occupation and income level.

### Short Form-36 (SF-36)

Participants’ HRQoL was assessed using the Short Form-36 (SF-36) [31]. The SF-36 provides an overall impression of HQoL, which encompasses various aspects of functioning and wellbeing. These aspects were divided into two different components, namely the physical component summary (PCS) and the mental component summary (MCS). Higher scores reflected better functioning and greater wellbeing. The SF-36 established acceptable reliability and validity with Malaysian respondents, with an internal consistency of 0.7 [32]. It was also found to correlate with other generic health surveys.

### Big Five Inventory (BFI)

The five major personality traits of participants were measured by the Big Five Inventory (BFI) [33]. This 44-item inventory measured the five personality characteristics of the Big Five Factors of personality, specifically extraversion, agreeableness, conscientiousness, neuroticism and openness. Participants were required to rate the extend that they agreed or disagreed concerning how true each of the 44 statements applied to them on a Likert scale of 1 to 5, with 1 indicating that they disagree strongly, and 5 being agreeing strongly with the statements given. The BFI showed high convergent validity with other personality questionnaires, such as the NEO [33], [34]. The BFI also demonstrated acceptable reliability and validity within the Malaysian context. The internal consistency of the BRI was generally 0.7 for each factor [35].

### Medical Outcome Study (MOS) Social Support Survey

Social support was measured using the Medical Outcome Study (MOS) Social Support Survey [36]. This survey consisted of items that assessed four different dimensions of social support, which were emotional/informational, tangible, affectionate, and positive social interaction, as well as an overall functional social support index. Emotional support is the kind of support that involves expression of love, care and empathy, whereas informational support include advice, information, guidance and feedback that is useful to the patient in solving their issues. Tangible support is the kind of support that involves physical aid or behavioural assistance, for example, helping the patient to see a doctor if needed. Affectionate support involves the expression of love and affection by others that are felt by the patients. Positive social interaction includes social integration, feeling like they belong and social companionship. The MOS Social Support Survey exhibited good internal consistency (Cronbach’s alpha = 0.93), and good validity [37].

### Data Analysis

Data were analysed using the Statistical Package for Social Sciences (SPSS) version 22.0. Descriptive statistics were used to analyse the demographic information gathered. Pearson correlation analysis was employed to investigate the relationship between variables and multiple linear regression analyses with enter method were conducted to assess the influence of personality and social support in relation to HRQoL in patients with CKD. Identical multiple regression analysis was performed for physical and mental HRQoL separately.

## Results

Table 1 shows the demographic profile of the current study. Participants consisted of 108 males (54.0%) and 92 females (46.0%) with the mean age of 59.3 years (SD 14.5). Most of the participants were of Malay ethnicity (66.5%), followed by Chinese (26.5%), Indian (6.5%) and others (0.5%). Out of the 200 participants, the majority of participants were currently in Stage 3 in their CKD progression. Diabetes was reportedly the cause of CKD for most participants.

**Table 1:** Patient characteristics with their frequency and percentage.

Patient Characteristics	n (%)
**Sex**	
Male	108 (54.0%)
Female	92 (46.0%)
**Age**	
18–39	23 (11.5%)
40–64	91 (45.5%)
65+	86 (43.0%)
**Ethnicity**	
Malay	133 (66.5%)
Chinese	53 (26.5%)
Indian	13 (6.5%)
Others	1 (0.5%)
**CKD Stages**	
Stage 2	4 (2.0%)
Stage 3	81 (40.5%)
Stage 4	63 (31.5%)
Stage 5	52 (26.0%)
**Cause of kidney disease**	
Diabetes Mellitus	111 (46.6%)
High blood pressure	70 (29.4%)
Kidney stones	2 (0.8%)
Lupus Nephritis	3 (1.3%)
Others	10 (4.2%)
Unknown	4 (1.7%)

The correlations between age, GFR, the Big Five personality traits, social support and the physical (PCS) and mental (MCS) components of the HRQoL are displayed in Table 2 along with the means and standard deviations. Pearson’s correlation was employed to explore the relationships between these variables. Older patients tended to rate poorer physical HRQoL. Higher GFR scores (earlier stages of CKD) were associated with better perceived physical and mental HRQoL. Patients reporting being more open to new experiences and more extraverted reported better physical HRQoL. Patients who perceive having higher affectionate social support also reported better physical HRQoL. Patients who reported being open, conscientious and extraverted correspondingly reported better mental HRQoL. Patients high in neuroticism tended to respond with poorer mental HRQoL. None of the social support components correlated significantly with neither physical nor mental HRQoL. Better physical HRQoL was associated with better mental HRQoL.

**Table 2:** Means, standard deviation (SD), and Pearson correlations (r) of the age, GFR, components of Big Five traits, social support and physical (PCS) and mental (MCS) health-related quality of life.

Variables	Mean	SD	PCS	MCS
**Age**	59.32	14.45	-.22**	-.13
**GFR**	27.29	15.16	.33**	.22**
**Openness**	30.76	5.17	.24**	.27**
**Conscientiousness**	34.09	4.25	.11	.27**
**Extraversion**	27.64	4.11	.22**	.28**
**Agreeable**	35.79	3.55	-.06	.01
**Neuroticism**	19.30	4.84	-.14	-.38**
**Emotional/Informational Social Support**	32.67	6.20	-.07	-.11
**Tangible Social Support**	17.30	3.09	-.13	-.10
**Affectionate Social Support**	12.79	2.67	.19**	.07
**Social Interaction Social Support**	12.56	2.18	-.11	.02

Note. Statistical significance:

*p < .05;

**p < .01

Hierarchical multiple regression analysis was then performed to investigate how much of the variance in physical HRQoL could be explained by personality and social support after controlling for age and GFR. The hierarchical model for physical HRQoL is summarised in Table 3. For physical HRQoL, age and GFR were entered in the first block, followed by openness, extraversion and affectionate social support in the second block. At Step 1, age and GFR accounted for 13% of the variance in physical HRQoL, Fch (2, 197) = 14.21, p < .001. Both age and GFR were significantly associated with physical HRQoL, such that being older was associated with poorer physical HRQoL, β = -.14, p = .043, age uniquely explained 2% of the variance. GFR also associated with HRQoL, such that earlier stages of CKD was associated with better physical HRQoL, β = .29, p < .001, GFR uniquely explained 8% of the variance. At Step 2, the addition of openness, extraversion and affectionate social support explained 6% of the variance over and above age and GFR, Fch (3, 194) = 4.84, p = .003. Both extraversion and affectionate social support were significantly associated with physical HRQoL. Being extraverted was associated with better physical HRQoL, β = .15, p = .035, extraversion uniquely explained 2% of the variance. Perceiving more affectionate social support was associated with better physical HRQoL, β = .15, p = .021, affectionate social support uniquely explained an additional 2% of the variance. Openness was not significantly associated with HRQoL. Overall, at Step 2, age, GFR, openness, extraversion and affectionate social support significantly accounted for 19% of the variance in physical HRQoL, F (5, 194) = 8.92, p < .001.

**Table 3:** Hierarchical regression model of physical health-related quality of life.

Variables	R^2^	ΔR^2^	B	SE	β	t
**Step 1**	.13	.13**				
Age			-.14	.07	-.14*	-2.03
GFR			.27	.06	.29**	4.23
**Step 2**	.19	.07**				
Age			-.13	.07	-.13	-1.91
GFR			.22	.06	.24**	3.46
Openness			.19	.21	.07	.90
Extraversion			.53	.25	.15*	2.12
Affectionate Social Support			.81	.35	.15*	-2.32

Note. Statistical significance:

*p < .05;

**p < .01

Another hierarchical multiple regression analysis was performed to investigate how much of the variance in mental HRQoL could be explained by personality after controlling for GFR. The model for mental HRQoL was summarized in Table 4. In terms of mental HRQoL, GFR were entered in the first block, followed by openness, conscientiousness, extraversion and neuroticism. At Step 1, GFR accounted for 5% of the variance in mental HRQoL, Fch (1, 198) = 9.81, p = .002. GFR was significantly associated with mental HRQoL, such that earlier stages of CKD was associated with better mental HRQoL, β = .22, p = .002, GFR uniquely explained 5% of the variance. At Step 2, the addition of openness, conscientiousness, extraversion and neuroticism explained 19% of the variance over and above GFR, Fch (4, 194) = 11.98, p < .001. Only neuroticism was significantly associated with mental HRQoL, such that higher neuroticism was associated with poorer mental HRQoL, β = -.36, p < .001, neuroticism uniquely explained 9% of the variance in mental HRQoL. Overall, at Step 2, GFR, openness, conscientiousness, extraversion and neuroticism significantly accounted for 24% of the variance in mental HRQoL, F (5, 194) = 11.98, p < .001.

**Table 4:** Hierarchical regression model of mental health-related quality of life.

Variables	R^2^	ΔR^2^	B	SE	β	t
**Step 1**	.05	.05**				
GFR			.18	.06	.22**	3.13
**Step 2**	.24	.19**				
GFR			.15	.05	.19**	2.86
Openness			.32	.18	.13	1.74
Conscientiousness			.17	.26	.06	-.67
Extraversion			.41	.23	.14	1.80
Neuroticism			-.93	.20	-.36**	-4.79

Note. Statistical significance:

*p < .05;

**p < .01

## Discussion

This study was conducted to explore the contributions of the Big Five personality traits and social support to HRQoL in patients with CKD. The findings revealed that both age and the progression of CKD were associated with HRQoL, such that older age and later stages of CKD were associated with poorer HRQoL. Two of the Big 5 personality traits, namely extraversion and neuroticism, were also associated with HRQoL. In relation to social support, we found that only affectionate social support had an associated with physical HRQoL.

The inverse relation between age and HRQoL was found in the current study, which was consistent with findings of previous studies [17], [18], [38]. However, it should not be interpreted that age causes poorer HRQoL per se. With the progression of age, the period of having CKD is also prolonged. With a longer duration of having the disease, there might be an accumulation of various other factors that could influence the progression of the disease and the wellbeing of the patient. Therefore, HRQoL is usually found to decline over time [38].

Various studies demonstrated the association between the progression of CKD and HRQoL [15], [38]. Similarly, our results also found that HRQoL is compromised with the decreased functioning of kidneys due to CKD, as measured by GFR. This also tied in with the previous point. Over time, if the patient does not seek appropriate treatment, CKD could progress into more advance stages of the disease. With increased deterioration of kidney function, various health and lifestyle changes have to be practiced in order to maintain functions of the kidney. In addition, it was argued that the knowledge of having the disease or being labeled could also influence HRQoL [17]. This was found in patients who were also having hypertension on top of CKD who reported poorer HRQoL despite hypertension being asymptomatic [17]. However, there are studies that found no association between changes in HRQoL and changes in GFR [11] [18], [39]. Therefore, it could be suggested that the association between HRQoL and GFR may also be influenced by other factors such as anemia, nutritional status, and albumin among others.

Extraversion was found to be significantly associated with physical HRQoL. This was consistent with findings of previous studies. Higher levels of extraversion were related to better health outcomes [40], [41], [42]. In addition, a study done on patients who recently had a kidney transplant found that higher extraversion was associated with better mental HRQoL [41]. Extraverted people tended to engage in more active coping mechanisms, and they preferred external stimulation [41]. Therefore, it could be argued that they may be easily distracted away from their disabilities.

Neuroticism was also found to significantly associate with poorer mental HRQoL. This finding supported studies finding an inverse relationship between neuroticism and perceived health [21], [40], [41]. These studies found that people with lower neuroticism traits tended to reported better health outcomes [40], better health perception [21], [42], [43], as well as both physical and mental HRQoL [16]. One reason for this could be that people with higher neuroticism traits tend to be more negative in the way they perceive the world. Therefore, they were likely to be hypervigilant towards negative stimuli that they encounter. It is no surprise then, that patients with higher trait neuroticism would be more preoccupied with their health symptoms, and complain about it more and consequently perceived poorer HRQoL [16], [41]. Moreover, healthy people without any medical problems with type of personality also tended to perceive poorer health [21].

There was no association found between conscientiousness, agreeableness and openness and HRQoL. This could be due to the fact that these personality factors do not influence HRQoL directly, but they are associated with other behaviours that may predict better health. This may be true for other personality traits, except for openness, whereby it was found to be not a correlate of self-rated health [40]. Higher levels of agreeableness, conscientiousness and extraversion were found to predict better self-rated health [40]. Conscientiousness was better associated with treatment adherence [42], [44] and therefore might have lead to improved HRQoL. These behaviors and outcomes might not necessarily translate into quality of life, as they might not be living their life as how they would like, and therefore perceived their quality of life to be significantly affected by their illnesses. This could suggest that how patients view their illness may play a bigger role in predicting HRQoL rather than patients’ personality traits.

Contradictory to the literature [26], [27], social support was not a significant predictor of HRQoL, except for affectionate social support, which was the expression of love and affection, was found to significantly predict physical HRQoL. Consistent with a recent study done on breast cancer patients, social support, specifically emotional and informational support as well as affectionate and positive social interaction support were more important in improving quality of life, rather than tangible social support [45]. Patients felt that emotionally focused support was better in helping them cope with high distressing events. However, not all social supports were perceived to be helpful [46], [47]. Certain behaviours that were thought to be supportive may be perceived as unhelpful to patients, such as overreactions, unhelpful advice, and tendency to treat the patient as an invalid among others [48]. Being overly concerned about the patient might also make them feel different and it would highlight their disability and sickness [49].

### Limitations and future research

The main limitation of this study was that it was a cross-sectional study, as such no causal inferences could be made between personality traits and social support and their prediction towards HRQoL. In addition, we did not use a kidney disease-specific measure of HRQoL, such as the Kidney Diease Quality of Life Instrument (KDQOL). However, various other studies have also employed the generic SF-36 to explore HRQoL in CKD patients. We also did not find any association between social support and HRQoL aside from affectionate social support. More qualitative exploration may provide answers to why we did not find this association.

Nevertheless, our findings emphasised the need for psychological assessment especially in terms of patients’ personality traits as they might influence HRQoL to a certain extent, especially neuroticism and extraversion. Our findings also suggest that patients perceived certain social support to be helpful, such as affectionate social support. Taking into account these factors in intervention may improve patients’ health outcome as well as their quality of life.

## Conclusion

Despite the limitations noted, the current study added to existing literature that investigated psychological factors and quality of life in CKD. Moreover, this study was among the first that explored personality traits predicting HRQoL in CKD within Malaysian context. Consistent with previous findings, we found that certain personality traits were significantly associated with HRQoL such neuroticism as well extraversion. In addition, affectionate social support was also found to be significantly associated HRQoL These were all important factors to take into account when designing intervention for patients with CKD in order improve their wellbeing and health outcomes. Future studies should look into more qualitative exploration, especially social support so to be able to explore what kind of social support patients feel to be useful and supportive in aiding their treatment effects, and also to see if certain personality traits might perceive different types of social support to be helpful versus non-helpful and what kind of effect will that have on quality of life.

## Supporting Information

S1 Information (SAV)
